# 1929. A Qualitative Analysis of COVID-19 Vaccination Intent and Recommendations to Increase Uptake Among Residents and Staff in Six Seattle Homeless Shelters

**DOI:** 10.1093/ofid/ofac492.1556

**Published:** 2022-12-15

**Authors:** Sarah N Cox, Nicholas Thuo, Julia H Rogers, Ashley A Meehan, Amy C Link, Natalie K Lo, Brian Manns, Constance E Ogokeh, Eric J Chow, Melissa A Rolfes, Emily Mosites, Morhaf Al Achkar, Helen Y Chu

**Affiliations:** University of Washington, Seattle, Washington; University Of Washington, Seattle, Washington; University of Washington, Seattle, Washington; Centers for Disease Control and Prevention, Atlanta, Georgia; University of Washington, Seattle, Washington; University of Washington, Seattle, Washington; Centers for Disease Control and Prevention, Atlanta, Georgia; Centers for Disease Control and Prevention, Atlanta, Georgia; Public Health - Seattle & King County, Seattle, Washington; Centers for Disease Control and Prevention, Atlanta, Georgia; CDC, Atlanta, Georgia; University of Washington, Seattle, Washington; University of Washington, Seattle, Washington

## Abstract

**Background:**

COVID-19 vaccines are important to mitigate severe disease in congregate settings, yet uptake remains lower among people experiencing homelessness (PEH) than in the general population. This study aimed to explain changes in COVID-19 vaccination intent over time and identify modifiable factors to improve vaccine acceptance among PEH.

**Methods:**

We utilized the Health Belief Model and 3Cs Model of Vaccine Hesitancy to develop a conceptual framework to explore factors that may influence COVID-19 vaccination intent among PEH. Between July 27 - October 15, 2021, we conducted semi-structured interviews (SSIs) and focus group discussions (FGDs) across six homeless shelters in Seattle-King County, Washington. Residents and staff aged 18 years and older were recruited through purposive sampling for SSIs and convenience sampling for FGDs. We captured retrospective information about perceptions of and intent to receive COVID-19 vaccines between March 2020 - August 2021. Thematic analysis was conducted using Dedoose.

**Results:**

We conducted 31 SSIs (25 residents and six staff) and eight FGDs with 43 residents. Participants reported that too much contradictory and changing information about COVID-19 vaccines led to confusion. Information deemed trustworthy (i.e., objective, honest, professional, and recommended by others) contributed to individual’s knowledge and in some cases changed their vaccination intent. Despite intention to vaccinate, participants reported barriers to COVID-19 vaccine access including availability, eligibility, appointments, and timeliness. While many intended to get vaccinated on their own, others were motivated by incentives and requirements. Participants presented recommendations to improve COVID-19 information content and dissemination, access, and incentives in shelter settings (Table 1).

**Table 1.**
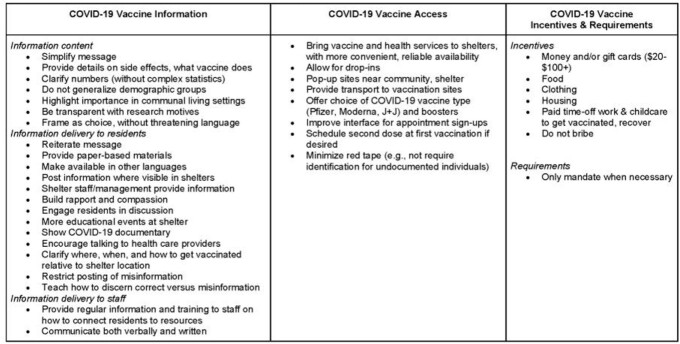
Recommendations for Interventions to Increase COVID-19 Vaccine Uptake in Shelter Settings

**Conclusion:**

COVID-19 vaccination strategies that are rooted in the voices and experiences of PEH are presented and can inform improved vaccine implementation among key stakeholders. Future research should test recommended strategies to determine feasibility and effectiveness in shelter settings.

**Disclosures:**

**Helen Y. Chu, MD, MPH**, Cepheid: Reagents|Ellume: Advisor/Consultant|Gates Ventures: Grant/Research Support|Merck: Advisor/Consultant|Pfizer: Advisor/Consultant.

